# Necrobotics: Biotic Materials as Ready‐to‐Use Actuators

**DOI:** 10.1002/advs.202201174

**Published:** 2022-07-25

**Authors:** Te Faye Yap, Zhen Liu, Anoop Rajappan, Trevor J. Shimokusu, Daniel J. Preston

**Affiliations:** ^1^ Department of Mechanical Engineering Rice University Houston TX 77005 USA

**Keywords:** biohybrids, bioinspiration, grippers, pneumatic actuators, soft robotics, spiders

## Abstract

Designs perfected through evolution have informed bioinspired animal‐like robots that mimic the locomotion of cheetahs and the compliance of jellyfish; biohybrid robots go a step further by incorporating living materials directly into engineered systems. Bioinspiration and biohybridization have led to new, exciting research, but humans have relied on biotic materials—non‐living materials derived from living organisms—since their early ancestors wore animal hides as clothing and used bones for tools. In this work, an inanimate spider is repurposed as a ready‐to‐use actuator requiring only a single facile fabrication step, initiating the area of “necrobotics” in which biotic materials are used as robotic components. The unique walking mechanism of spiders—relying on hydraulic pressure rather than antagonistic muscle pairs to extend their legs—results in a necrobotic gripper that naturally resides in its closed state and can be opened by applying pressure. The necrobotic gripper is capable of grasping objects with irregular geometries and up to 130% of its own mass. Furthermore, the gripper can serve as a handheld device and innately camouflages in outdoor environments. Necrobotics can be further extended to incorporate biotic materials derived from other creatures with similar hydraulic mechanisms for locomotion and articulation.

## Graphical abstract

This work introduces a new area of research where biotic materials are used directly as actuators with minimal fabrication steps required. Taking advantage of the spider's hydraulic mechanism for leg extension, a fully functional pneumatic necrobotic gripper is fabricated in a single step by inserting a needle into the body of a deceased spider.

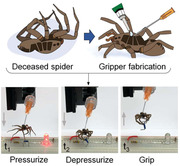

## 1 Introduction

By observing organisms that have been refined through millennia of evolution, scientists and engineers have developed capabilities ranging from surface wettability modification based on lotus leaves and Namib beetles^[^ ^1^, ^2^, ^3^ ^]^ to adhesion mechanisms that emulate gecko toes,^[^ ^4^, ^5^, ^6^ ^]^ and even sensing for smart materials by mimicking the humidity‐sensitive pine cone and the color‐changing chameleon.^[^ ^7^, ^8^, ^9^, ^10^, ^11^ ^]^ Researchers have also drawn inspiration from locomotion of terrestrial animals (e.g., cheetahs, snakes, and insects) and aquatic creatures (e.g., starfish, jellyfish, and octopuses) to guide the design of robots and actuators.^[^ ^12^, ^13^, ^14^, ^15^, ^16^, ^17^, ^18^, ^19^, ^20^, ^21^ ^]^ Many of these creatures interact with unknown terrain and react to their environment with the help of motion‐ or force‐limiting compliant bodies^[^ ^22^ ^]^ and other intrinsic material and structural advantages. Leveraging properties and mechanisms similar to those generated by nature can enable engineered systems with entirely new capabilities or advantages; this field of inquiry is referred to as bioinspired or biomimetic design.^[^ ^23^ ^]^

Going beyond inspiration, biohybrid systems combine natural or engineered cells and tissues with artificial or inanimate components to generate biocompatible robots and actuators at small length scales capable of producing considerable forces.^[^ ^24^ ^]^ The biological material used in these systems ranges from the microscale (e.g., cardiomyocytes, skeletal muscle tissues, and motile bacteria)^[^ ^25^, ^26^, ^27^, ^28^, ^29^, ^30^ ^]^ to the macroscale (e.g., the Venus flytrap and cockroaches).^[^ ^31^, ^32^, ^33^ ^]^ Park et al. recently combined biohybrid and biomimetic systems to create a tissue‐engineered swimming ray by printing rat cardiomyocytes that can be stimulated optically to generate downward contractions in an elastomeric body inspired by the ray.^[^ ^28^ ^]^ While the use of living biological materials in biohybrid systems has paved the way for innovative new research in recent decades, biotic materials—nonliving materials derived from living organisms, such as silk, leather, wood, and wool—have been used by humans even before the advent of modern science. The useful properties of biotic materials, refined by nature over time, eliminate the need to artificially engineer these materials, exemplified by our early ancestors wearing animal hides as clothing and constructing tools from bones. We propose leveraging biotic materials as ready‐to‐use robotic components in this work due to their ease of procurement and implementation, focusing on using a spider in particular as a useful example of a gripper for robotics applications.

Spider‐inspired devices and systems have garnered a particularly high level of interest in recent years due to the unique mechanism through which spiders control the motion of their legs: by applying hydraulic pressure. Spider legs do not have antagonistic muscle pairs; instead, they have only flexor muscles that contract their legs inwards, and hemolymph (i.e., blood) pressure generated in the prosoma (the part of the body connected to the legs) extends their legs outwards.^[^ ^34^ ^]^ When a spider dies, pressure no longer opposes the flexor muscles, causing the spider's legs to curl inward as commonly observed among deceased household spiders.

Researchers have explored a range of designs based on spiders, from rigid and hard hexapod robots inspired by the gait of spiders to soft grippers inspired by the actuation mechanism and architecture of spider joints.^[^ ^35^, ^36^, ^37^, ^38^, ^39^, ^40^ ^]^ This fascination with spiders is not unwarranted; a spider's body relies on a synergy of both rigid and soft components that allows them to navigate through harsh environments with the help of their compliant geometric design and fluidic actuation.^[^ ^22^ ^]^ For example, the compactness enabled by the hydraulic system, as compared to antagonistic muscle pairs, has allowed larger muscles for flexion which facilitate more efficient and powerful movements required for weaving webs and hunting prey.^[^ ^41^ ^]^ This high power density enables the spider's ability to produce explosive movements and serves as an inspiration to design compact and high‐power actuators. For example, Lin et al. designed a high‐speed soft gripper based on a snap‐through instability by using a prestreched rubber band (the “flexor muscle”) at the joint of a gripper and pneumatic control (the “hemolymph pressure”) to extend the gripper.^[^ ^35^ ^]^ Kellaris et al. mimicked the actuation and functional characteristics of the spider joint by developing a soft electrohydraulic joint that leverages mechanisms from linear Peano‐HASEL actuators and rotational pouch motors, an elastic hinge at the joint to generate the restoring force, and a stiff exoskeletal layer to provide rigidity, leading to a lightweight jumping robot.^[^ ^37^ ^]^ The body of work concerning spider‐inspired technologies and designs even includes one publication on the control of a live spider using electrical stimulation;^[^ ^42^ ^]^ however, aside from spider silk and molted spider exoskeletons,^[^ ^43^, ^44^ ^]^ the incorporation of biotic materials derived from the spider body itself has not yet been explored.

**Figure 1:**
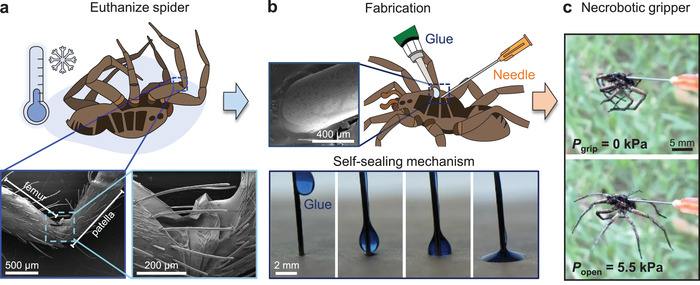
Fabrication of the necrobotic gripper. a) A spider is euthanized by application of cold temperature. The patellofemoral joint of the spider was visualized with a scanning electron microscope (SEM) to show the articular membrane at the joint with the stroller sun shade geometry. b) A 25‐gauge hypodermic needle is inserted into the prosoma of the spider and sealed with glue. The SEM image shows the glue forming an airtight seal around the needle and the cuticle (or exoskeleton). We performed the same procedure with blue‐dyed glue on a similar needle to illustrate the self‐sealing mechanism. c) After the glue has cured, a syringe (or any suitable pressure source) is connected to the needle, completing the fabrication of the necrobotic gripper.

In this work, we employ the intact body of an inanimate biological creature (a dead spider) as a ready‐to‐use biotic actuator (**Figure** **1**) in the first step toward an area of research we call “necrobotics.” Our strategy contrasts with bioinspired approaches in which researchers look to the spider's physical morphology for design ideas that are subsequently implemented in complex engineered systems, and also differs from biohybrid systems in which live or active biological materials serve as the basis for a system, demanding careful and precise maintenance.^[^ ^24^, ^27^ ^]^ We repurposed the cadaver of a spider to create a pneumatically actuated gripper that is fully functional following only one simple assembly step, allowing us to circumvent the usual tedious and constraining fabrication steps required for fluidically driven actuators and grippers. For instance, PneuNet grippers typically require 3D‐printed molds with limited resolution and a lower bound on length scale, internal geometries that must be accounted for by casting and assembling several distinct components, and elastomers that take hours or days to cure.^[^ ^15^, ^45^, ^46^, ^47^, ^48^ ^]^ Our ready‐to‐use pneumatically actuated necrobotic gripper is able to lift an object 1.3 times its own weight, exerts a peak gripping force of 0.35 mN, and can withstand 700 actuation cycles before the onset of degradation. We also demonstrated the necrobotic gripper's ability to lift objects with varying geometries, volumes, and masses, and we showed that untethered, handheld actuation is possible. Our necrobotic gripper can camouflage with its surroundings due to the patterns and texture on its body, and furthermore, it naturally biodegrades at the end of its lifetime, reducing the amount of waste generated compared to traditional artificial gripper actuators.^[^ ^49^ ^]^

## 2 Results

### 2.1 Necrobotic Gripper Fabrication Procedure

The raw biotic material (i.e., the spider cadaver) was obtained by euthanizing a wolf spider (of the family *Lycosidae*) through exposure to freezing temperature (approximately ‐4 °C) for a period of 5–7 days (Figure 1a).^[^ ^42^, ^50^ ^]^ Scanning electron microscope (SEM) images of the patellofemoral joint (insets of Figure 1a) show the articular membrane (resembling the folding geometry of a stroller sun shade) that has inspired the design of many soft flexible joints.^[^ ^38^, ^51^ ^]^

The fabrication of a necrobotic gripper requires only one simple step: inserting a needle into the prosoma region of a deceased spider and fixing the needle to the spider's body with glue to form a hermetic seal (Figure 1b). The droplet of glue navigated to the needle–spider interface without any external manipulation due to the minimization of energy in three phases: First, the droplet was administered on the side of the needle shaft and adhered to the needle to minimize its surface energy; second, it traveled down the shaft of the needle until it contacted the surface of the spider cuticle (or exoskeleton) by gravity; and finally, when in contact with the cuticle of the spider, it formed a meniscus along the needle–cuticle interface, again due to minimization of surface energy, thereby creating an airtight seal as it cured. The entire process, from inserting the needle to the setting of the glue to create a fully operational gripper, can be performed in approximately ten minutes. We recreated this self‐sealing mechanism (Figure 1b) with a drop of glue (dyed blue) on a needle inserted into paper (mimicking the spider cuticle) to illustrate the dynamic energy minimization of the glue droplet as it spreads across the surface and forms a meniscus with the needle. The needle insertion point on the spider was imaged with SEM (inset of Figure 1b) to show the seal between the needle and cuticle. Snapshots of the necrobotic gripper fabrication procedure are provided in Figure S1 of the Supporting Information.

We chose the prosoma (i.e., the anterior part of the spider body where the legs are attached) as the insertion site for two reasons: i) the hydraulic pressure for spider leg movement is generated in the prosoma;^[^ ^34^, ^41^, ^52^ ^]^ and ii) the exoskeleton of the prosoma is more rigid than that of the abdomen.^[^ ^34^, ^53^ ^]^ Because the spider legs only contain flexor muscles, after death, the spider legs curl toward the body due to the lack of hydrostatic pressure (generated by muscles in the prosoma when the spider is alive) acting to extend the legs.^[^ ^34^, ^41^, ^52^ ^]^ After the glue sets (after approximately ten minutes for the cyanoacrylate glue used in this work; see Supporting Information), a syringe (or other suitable pressure source) is attached to the Luer lock end of the hypodermic needle, completing the fabrication process of the necrobotic gripper (Figure 1c). When no pressure is applied to the necrobotic gripper, the legs remain flexed inwards, which we refer to as its neutral state corresponding to “gripping;” in contrast, when pressure is introduced via the syringe, the legs actuate by extending outward and the gripper opens.

### 2.2 Force versus Displacement Characterization of Necrobotic Gripper

A spider extends each leg by actively contracting muscles in the prosoma to increase its internal hydraulic pressure. For the necrobotic gripper shown in our work, the external pneumatic pressure source replaces the role of active hemolymph pressure regulation in the spider, actuating the legs when the pressure within the spider increases above atmospheric pressure (Figure 1c). Pressurizing and depressurizing result in opening and closing, respectively, of all of the spider's legs simultaneously, effectively acting as a gripper.

**Figure 2:**
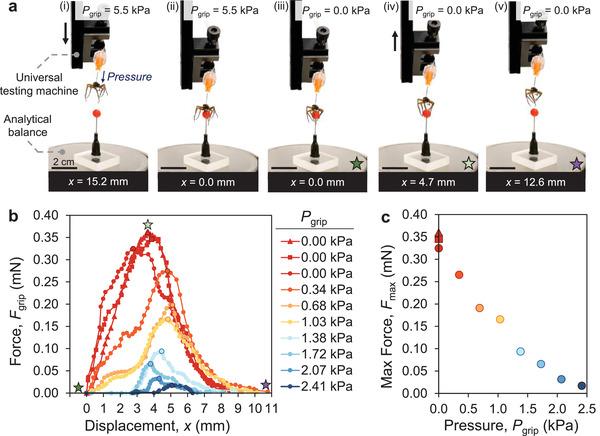
a) Experimental sequence employed to determine the gripping force of the necrobotic gripper by using a universal testing machine to control the displacement and an analytical balance to measure the gripping force of the gripper. a‐i) The necrobotic gripper is pressurized to open, and approaches the “weight” (i.e., the red bead). a‐ii) After the necrobotic gripper almost contacts the weight, a‐iii) the necrobotic gripper is depressurized to grip onto the weight. a‐iv) The universal testing machine raises the necrobotic gripper at a constant, quasi‐static velocity, and the analytical balance displays the effective gripping force exerted on the weight by the necrobotic gripper. a‐v) The gripping force is zero when the necrobotic gripper is no longer in contact with the weight. b) The gripping force for the same spider was characterized at eight different gripping pressures, *P*_grip_, and plotted against displacement. We performed three trials at *P*_grip_ = 0 kPa and found that, while minor variability in the gripping force appears at a given pressure, the overall performance remains consistent and follows a monotonic trend (higher *F*_grip_ at lower *P*_grip_). The colored stars in (a) correspond to the force at each displacement in (b) to provide additional context for the characterization process. c) The maximum gripping force exerted by the necrobotic gripper at each gripping pressure is plotted, showing that an increase in gripping pressure results in a decrease in maximum gripping force.

We characterized the gripping force of the necrobotic gripper using a setup consisting of a universal testing machine and an analytical balance (Figure S2, Supporting Information). We affixed the necrobotic gripper onto a clamp where the displacement, *x*, is controlled by the universal testing machine. We placed a customized “weight” to be gripped by the necrobotic gripper on the analytical balance to measure the effective gripping force. The weight consists of a red cellulose acetate bead (with a diameter of 3.03 mm) attached to a thin metal shaft to elevate the bead off the surface of the analytical balance for ease of gripping, and the bead‐shaft structure was glued to a heavy acrylic base for additional stability and to ensure that it did not lift off of the analytical balance during testing. A pressure gauge and a syringe were connected to the necrobotic gripper by a tube to regulate the internal pressure and study the effects of gripping pressure on gripping force. We show a time‐lapse of the force characterization procedure in **Figure** **2**a. The necrobotic gripper was pressurized to open (*P*_grip_ = 5.5 kPa gauge) and lowered by the universal testing machine until the body of the necrobotic gripper was almost in contact with the red bead; the displacement and mass readings on the universal testing machine and analytical balance were then zeroed (Figure 2b). The typical pressure in the legs of a resting spider ranges from 4.0 kPa to 6.1 kPa,^[^ ^41^ ^]^ so we chose the opening pressure of 5.5 kPa within this range to avoid damaging the necrobotic gripper while also ensuring that the gripper leg span has enough clearance to grasp the object. We measured the gripping force when the necrobotic gripper is closed, i.e., in its neutral state (*P*_grip_ = 0 kPa). The universal testing machine raised the necrobotic gripper at a rate of 0.2 mm/s for quasi‐static gripping, and a camera setup was used to simultaneously capture the mass and displacement readings on the analytical balance and the universal testing machine displays. The negative mass reading (as a result of the lifting force exerted by the gripper on the weight) from the analytical balance was converted to force, and the force versus displacement plot of the necrobotic gripper was determined for an array of gripping pressures, shown in Figure 2b; the necrobotic gripper is able to exert forces on the order of hundreds of micronewtons. In contrast to typical pressure‐driven grippers that rely on continuous power input to remain in a gripping state,^[^ ^45^, ^48^, ^54^ ^]^ our necrobotic gripper only requires power (pressure) to open, and can remain in a gripping state indefinitely without an external power supply. As the gripping pressure increases, the gripping force of the necrobotic gripper decreases because the pressure opposes the flexor muscles of the spider (which exert the gripping force on the object being grasped). The necrobotic gripper achieves a maximum gripping force of 0.35 mN when it is in its fully closed state (0 kPa). The results show that we are able to modulate the gripping force by adjusting *P*_grip_, attaining gripping forces as low as 0.02 mN (Figure 2c). We also performed this experiment with beads of different sizes to show the effects of varying geometric parameters on the gripping force, and we found that the maximum gripping force increases with bead size (Figure S10, Supporting Information). The maximum gripping forces determined for each of the three bead sizes are within an order of magnitude (≈1 mN).

### 2.3 The Effect of Pressure on Joint Angle

**Figure 3:**
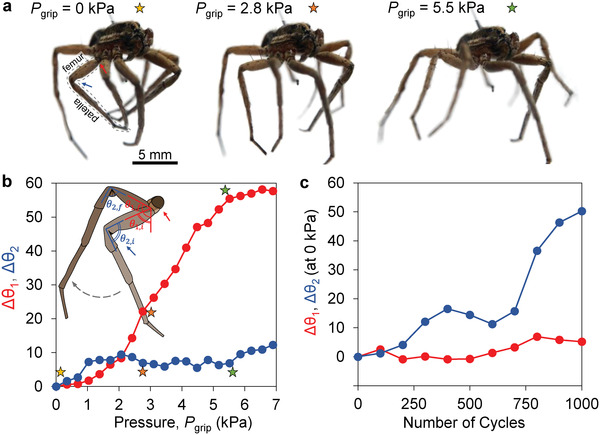
a) Joint angles of the spider leg in the gripper's neutral state and at two elevated actuation pressures. The leg that we characterized is outlined with dashes, and the two joints, (i) trochanter and (ii) patellofemoral, are indicated with red and blue arrows, respectively. b) We determined the change in joint angle for both joints, Δ*θ*, with respect to the joint angle in the neutral state (0 kPa) for different *P*_grip_. The data on the plot emphasized with colored stars correspond to the joint angles in (a). c) Cyclic testing was performed by opening and closing the necrobotic gripper for 1000 cycles, and the change in the neutral state joint angle with respect to the neutral state joint angle at the zeroth cycle was measured every 100 cycles.

We also characterized the joint angle of the necrobotic gripper at each gripping pressure. We focused on one of the spider legs and two of the joints: i) the trochanter joint, *θ*_1_, and ii) the patellofemoral joint, *θ*_2_, denoted with red and blue arrows, respectively, in **Figure** **3**a. The first image in Figure 3a shows the necrobotic gripper in its neutral closed state, where the gripper is not pressurized. The subsequent images show the change in joint angles at *P*_grip_ of 2.8 and 5.5 kPa. We define the trochanter joint angle as the angle of the femur with respect to the vertical and the patellofemoral joint angle as the angle between the femur and patella. We recorded images of the spider for actuation pressures ranging from 0  to 6.9 kPa (6.9 kPa = 1 psi) and plotted the change in angle at each joint, Δ*θ*, which is the difference between the final joint angle, *θ*_f_, and the initial joint position at 0 kPa, *θ*_i_. The changes in joint angle for the trochanter joint (red) and patellofemoral joint (blue) are represented by Δ*θ*_1_ and Δ*θ*_2_, respectively. We observed a significant change in joint angle (Δ*θ*_1_ > 50°) for the trochanter joint and a smaller change in joint angle (Δ*θ*_2_ < 12°) for the patellofemoral joint at high actuation pressures.

### 2.4 Cyclic Testing of Necrobotic Gripper

To study the durability of the necrobotic gripper, we conducted cyclic testing of gripper actuation by opening (*P*_grip_ = 5.5 kPa) and closing (*P*_grip_ = 0 kPa) the gripper for 1000 cycles, during which we measured the change in both the trochanter and patellofemoral joint angles relative to the initial (zeroth cycle) joint angles at 0 kPa. Despite having the least change in joint angle after being pressurized for gripping, the patellofemoral joint experienced the most change in joint angle at 0 kPa after 1000 cycles (Δ*θ*_2_ ≈ 50°), possibly indicating degradation of the joint. Using scanning electron microscopy (SEM), we observe that after 1000 cycles of actuation, cracks appear on the articular membrane at the patellofemoral joint (Figure S5, Supporting Information), which could be due to dehydration, resulting in the membrane becoming more brittle and susceptible to mechanical fracture (Figure S9, Supporting Information). Conversely, the trochanter joint, which exhibited the larger change in joint angle upon pressurization, had significantly less change in joint angle at 0 kPa after 1000 cycles (Δ*θ*_1_ ≈ 7°). These results indicate that the material properties of the trochanter joint, *θ*_1_, could be more durable and withstand higher pressures while undergoing large deformations as compared to the patellofemoral joint, *θ*_2_. We also performed X‐ray photoelectron spectroscopy (XPS) on the legs of the spider one hour after and seven days after euthanasia to characterize the change in chemical composition of the legs. From the XPS results, we determined the difference in elemental percentage of the two cases (an hour following euthanasia and seven days after euthanasia) to be less than 2% for the leg, and results from high‐resolution XPS spectra show a difference of less than 6% for the chemical bonding between the two cases, indicating no major changes in chemical structure that could affect the gripper's functionality (Figures S6 and S7, Supporting Information).

### 2.5 Functionality of the Necrobotic Gripper

**Figure 4:**
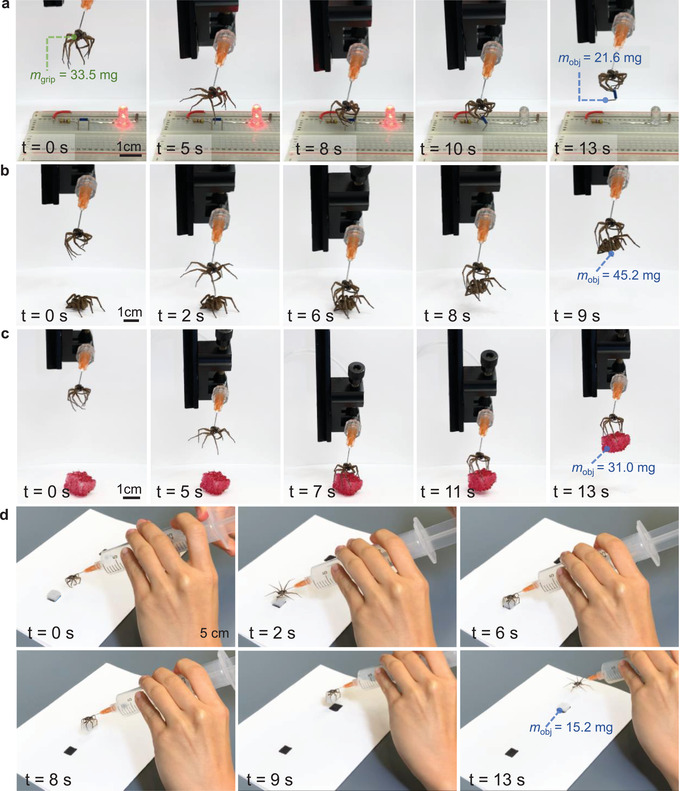
We highlight the ability of the necrobotic gripper to grasp various objects with different masses, *m*_obj_, and geometries. a) We show a closed circuit (LED on) which was then disconnected (LED off) when the necrobotic gripper removed a jumper wire that was affixed to the electric breadboard. The jumper wire is 0.64 times the mass of the necrobotic gripper and requires additional force to overcome friction when removing it from the breadboard. b) The necrobotic gripper also successfully grasps a spider which is 1.34 times the gripper's mass and has an irregular shape. c) We demonstrate that the necrobotic gripper can pick up objects with larger volumes by picking up a block of red‐dyed polyurethane foam (2.6 times the volume and 0.92 times the mass of the gripper). d) Furthermore, we show an untethered configuration for the necrobotic gripper by directly connecting a handheld pressure source to the needle of the necrobotic gripper, thereby converting it into a portable device. The handheld necrobotic gripper is able to transport a white polyurethane foam block (0.45 times the mass of the necrobotic gripper) from one location to another, and is not tethered to bulky control infrastructure.

We demonstrated the ability of the necrobotic gripper to grasp a variety of objects. Using a setup similar to the force‐versus‐displacement characterization experiment, we controlled the displacement in the vertical direction with the universal testing machine and regulated the pressure with a pressure gauge and a syringe. We used the same necrobotic gripper for all of the demonstrations shown in **Figure** **4**; the mass of this necrobotic gripper, *m*_grip_, was 33.5 mg.

We first showed that the necrobotic gripper is able to pick up slender objects that are semi‐affixed to a surface in Figure 4a. We constructed a circuit to power an LED with an additional jumper wire that acts as a switch. We pressurized the necrobotic gripper while simultaneously lowering it to approach the jumper wire (Movie S1, Supporting Information). Once the necrobotic gripper was in close proximity with the wire, we depressurized the necrobotic gripper to its closed state (*P*_grip_ = 0 kPa) to grip the wire, after which we raised the gripper with the universal testing machine. The necrobotic gripper overcame friction between the jumper wire and the breadboard to disconnect the closed circuit, turning off the LED.

We then demonstrated the necrobotic gripper's ability to grasp a brittle object with an irregular shape and a mass greater than the gripper itself (Figure 4b). For this demonstration, we repeated the operating procedure used in the demonstration described above and showed that the necrobotic gripper successfully picks up a large spider that is 1.3 times heavier than the mass of the necrobotic gripper itself (Movie S2, Supporting Information). Typical soft pneumatic grippers and particle‐jamming‐based grippers have been shown to be capable of handling fragile objects with irregular shapes (e.g., an egg); however, the objects are often large (≈1–10 cm) and structurally robust.^[^ ^46^, ^48^, ^54^, ^55^, ^56^, ^57^ ^]^ The lower limit of the size of these soft grippers is constrained by the fabrication process, which often makes them too large to grip objects at smaller length scales that are also brittle in nature. We note that there has been work on small, soft magnetic grippers and also biohybrid microtweezers; however, they require bulky control infrastructure or precise culture media conditions to function.^[^ ^25^, ^58^ ^]^

Additionally, we show that the necrobotic gripper was able to pick up a polyurethane foam block with a volume 2.6 times larger than the volume of the necrobotic gripper (Figure 4c). We hypothesize that the necrobotic gripper may rely on adhesive properties to pick up the block only using the distal part of the legs (Movie S3, Supporting Information), without fully grasping the object as seen in the first and second demonstrations with the jumper wire and inanimate spider. We infer that the microscopic hairs (setae) surrounding the leg of the spider (Figure S3, Supporting Information) play a role in enhancing the gripping force on the surface of a rough object through dry adhesion, which may also be advantageous for picking up objects with smooth surfaces.^[^ ^59^ ^]^ These three demonstrations highlight the capabilities of the necrobotic gripper to handle various geometries and even grip objects that are greater than its own mass and volume.

In addition to the demonstrations described above, we performed untethered actuation of the necrobotic gripper by connecting a syringe directly to the needle of the necrobotic gripper (Figure 4d). In doing so, we created a handheld necrobotic gripper that is untethered with multiple degrees of freedom, akin to a reach extender, in contrast to being constrained to a bulky system of control infrastructure. We show in Movie S4 (Supporting Information) the handheld necrobotic gripper being used to transport a polyurethane foam block from an initial location to another predetermined location. We also note that, as soon as the syringe is pressurized, the necrobotic gripper achieves a full range of motion in less than one second. Handheld actuation of our necrobotic gripper will allow for outdoor applications for gathering small and brittle samples or capturing small biological creatures (e.g., a fly) discreetly in nature by camouflaging in the environment as a result of the spider's innate surface pattern and texture (Figure S4, Supporting Information).^[^ ^60^ ^]^

### 2.6 Scaling Analysis of Spider Weight to Gripping Force

**Figure 5:**
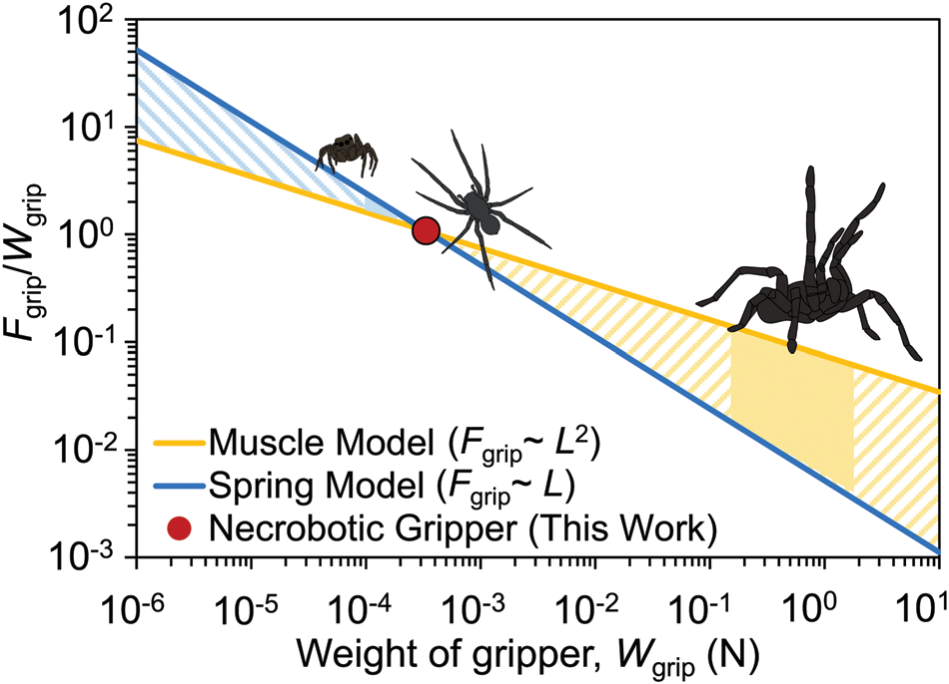
Scaling analysis of the gripper force compared to the weight of the gripper. We show a range of potential spider masses from 0.1 mg to 1 kg and predict the gripping force to gripper weight ratio. We present two models: (i) a muscle model where the gripping force, *F*_grip_, scales with *L* ^2^, and (ii) a spring model where the gripping force, *F*_grip_, scales with *L*.

To estimate the gripping force for necrobotic grippers made from spiders of different sizes, we performed a scaling analysis to understand the effect of spider weight on gripping force based on the results from our work (**Figure** **5**). To predict the ratio of gripping force, *F*_grip_, to the weight of the necrobotic gripper, *W*_grip_, for a given spider used to construct the necrobotic gripper, we hypothesized that the gripping force, exerted by the elastic inward flexing of the legs, falls in a region between two limiting cases: (i) application of force via contracting muscles,^[^ ^61^ ^]^ where *F*_grip_ scales as the characteristic length squared, *L* ^2^; and (ii) application of force via linear springs,^[^ ^62^ ^]^ where *F*_grip_ scales linearly with the characteristic length, *L*, of the gripper. The muscle‐type scaling is used to estimate forces in biological systems ranging from microscale to macroscale organisms, while the linear‐spring‐type scaling is typically used to estimate forces for elastic mechanisms.^[^ ^61^, ^63^ ^]^ We determined the scaling of the force‐to‐weight ratio by assuming that the characteristic length of the spider follows an isometric scaling law, in which *L* ∼ *W*_grip_^1/3^; using this scaling law, we converted between the characteristic length and weight.^[^ ^63^, ^64^ ^]^

The blue curve shows how the ratio of gripping force to the weight of necrobotic gripper scales with the weight of the necrobotic gripper for the spring scaling model, where *F*_grip_/*W*_grip_ ∼ *W*_grip_^−2/3^_,_ and the yellow curve shows the scaling of *F*_grip_/*W*_grip_ ∼ *W*_grip_^−1/3^ for the muscle scaling model. We fit these two curves through a point corresponding to the weight of the necrobotic gripper based on the wolf spider used in this work (*W*_grip_ = 0.000335 N) and the maximum gripping force achieved with this gripper (*F*_grip_ = 0.00036 N), and we determined the two relevant constants, *k*_spring_ and *k*_muscle_, to be 0.0052 N^2/3^ and 0.0747 N^1/3^, respectively.

The two curves encompass a region that predicts the range of expected *F*_grip_ to *W*_grip_ ratios based on the body weight of the spider used to construct a necrobotic gripper. The spider used in our work exerts a gripping force approximately equal to its own weight. From the scaling analysis, we predict that a smaller spider could hold more weight relative to its bodyweight (e.g., the jumping spider of the family *Salticidae*, which has a mass of 10–25 mg, would be able to exert a gripping force of over 200% its own weight). In contrast, a larger spider would not be able to hold as much weight relative to its bodyweight (e.g., the Goliath birdeater tarantula of the family *Theraphosidae*, which has a mass of ≈200 g, would only be able to grasp an object approximately 10% its own weight).

## 3 Discussion

The concept of necrobotics proposed in this work takes advantage of unique designs created by nature that can be complicated or even impossible to replicate artificially. We repurposed the body of a dead spider and created a gripper that harnesses the mechanism of spider movement. This concept builds on prior work that has paved the way for utilization of biotic materials, including i) using a mushroom for its umbrella geometry, porous matrix, and hydrophilic fibrous stipe to generate steam from solar energy,^[^ ^65^ ^]^ ii) extracting mucilage from flax seeds as an eco‐friendly drag reducing polymer,^[^ ^66^ ^]^ and, as is the case in many of our daily lives, iii) using leather from animal hides to create durable clothing, bags, and car seats.

In contrast to our necrobotic gripper based on biotic material, biohybrid systems exploit living biological cells and tissues to provide actuation and power artificial devices. Both systems, however, face similar limitations related to the longevity of the biomaterial used.^[^ ^24^ ^]^ We note that the functional duration of the necrobotic gripper was approximately two days after the death of the spider due to dehydration, which resulted in the joints becoming more brittle and susceptible to mechanical fracture with time. We hypothesize that we could extend the lifetime of the necrobotic gripper by applying a thin layer of vapor impermeable coating on the body of the spider to mitigate water loss. We performed preliminary experiments by coating an inanimate spider in a layer of beeswax and comparing the change in mass of the coated spider over a period of 10 days to an uncoated spider to support our hypothesis that coatings can prevent dehydration. Our results show that the uncoated spider had a 17× greater decrease in mass compared to the coated spider (Figure S9, Supporting Information). This initial finding warrants additional studies on different coating materials (e.g., application of oil or dip coating a layer of vapor‐impermeable polymer or wax on the exoskeleton) that can maintain flexibility in the joints while preventing dehydration, thereby prolonging the lifetime of the necrobotic gripper.^[^ ^67^, ^68^ ^]^ Because our necrobotic gripper is ultimately pressure‐driven, a hydraulic‐based actuation scheme that relies on the hemolymph itself could be realized if water loss could be prevented. To demonstrate that there should not be significant changes in hemolymph chemical properties over time that would affect the functionality of the necrobotic gripper, we characterized the chemical composition of the hemolymph one hour after euthanasia and seven days after euthanasia using XPS and found no significant changes in the elemental composition and chemical bonding (Figures S6 and S8, Supporting Information). We also note that there are currently no clear guidelines in the literature regarding ethical sourcing and humane euthanasia of spiders.^[^ ^69^ ^]^ We procured our spiders from a scientific product supplier and followed euthanasia methods presented in prior work. However, available guidelines for most spider euthanasia methods are tailored to prevent tissue damage for histological examination;^[^ ^70^ ^]^ future work could include characterization of the effects of different euthanasia methods, specifically motivated by improving the longevity of the necrobotic gripper. The present lack of information warrants further studies to establish clear regulations and instruction on the procurement of spiders and humane methods to euthanize them.^[^ ^71^, ^72^, ^73^ ^]^

Studying the gripping force for spiders of different sizes or species could lead to a more refined understanding of the relationship between spider size and gripping force. Future work could also include exploration of reinforcement methods that can be used to enhance the gripping force of the necrobotic gripper to fully take advantage of the gripper's small size and geometrical compliance. To realize an intelligent system, integrated sensing could potentially be interfaced with the necrobotic gripper to allow for autonomous gripping actuation. The use of low‐profile soft (even biodegradable) sensors that could provide force sensing capabilities will allow for the gripper to autonomously detect the target object upon contact by incorporating a resistive or piezoelectric sensor or the distance from the target object using a capacitive proximity sensor which will provide feedback to the pneumatic controller for actuation.^[^ ^74^, ^75^, ^76^, ^77^, ^78^, ^79^ ^]^ Additional levels of intelligence can be incorporated into our system in the future for autonomous navigation, situational awareness, and object detection. These embodiments could potentially allow the necrobotic gripper to handle small and brittle objects and also discreetly capture small biological creatures for sample collection in real‐world scenarios.

## 4 Conclusion

In this work, we complement the concepts of bioinspired design and biohybrid systems by introducing a new area of research which we called “necrobotics.” The design and fabrication of hard robots often entails intricate and tedious processes. The rise of soft robotics has successfully circumvented some of these complex fabrication requirements;^[^ ^48^, ^80^, ^81^, ^82^ ^]^ necrobotics, however, bypasses bulk of the fabrication process altogether by incorporating biotic materials as ready‐to‐use robotic components—in this case, we repurposed a dead spider as a necrobotic gripper. We show that the necrobotic gripper is able to grip objects that i) are delicate, ii) have irregular geometries, and iii) are of larger mass. We can also modulate the applied gripping force by varying the gripping pressure of the necrobotic gripper for applications which require forces on the order of tens to hundreds of micronewtons. Because the bulk of our necrobotic gripper is made of biodegradable materials, it will not significantly contribute to waste streams, and the concept of necrobotics could play a role in inspiring more sustainable fabrication of actuators to reduce the accumulation of technological waste. Moreover, by using biotic material as the actuator, we can take advantage of the inherent properties of the material, such as biodegradability and camouflaging capabilities, to potentially deploy them in nature to grip small and delicate samples in an unobtrusive and ecofriendly manner. Our work here presents the first step in this new avenue of research, which we expect will extend to locomotion of necrobots by independently actuating each leg of the spider, as well as the use of biotic materials derived from other creatures with similar hydraulic characteristics (e.g., *Patu digua* for microscale manipulation and whip scorpions for high‐speed articulation).

## 5 Experimental Section

### Scanning Electron Microscopy

The patellofemoral joint of the spider and the hermetic sealing of the hypodermic needle shown in Figure 1b were imaged using scanning electron microscopy (SEM). A single spider leg and the spider prosoma with the inserted and glued needle were taped down onto a piece of paper with an acrylic adhesive (Scotch Double Sided Tape, 6137H‐2PC‐MP). The spider leg and body were then sputtered with a 10 nm thick titanium coating that acts as an adhesion layer and a 20 nm thick coating of gold to improve the conductivity of the spider surface. The sputtering processes were conducted with an AJA ATC Orion 8 Sputtering System, with direct current (DC) plasma at a maximum power of 50 W. A field emission scanning electron microscope (FEI Quanta 400 ESEM FEG) was then used to take images with the secondary electron detector at an accelerating voltage of 10 kV.

### X‐Ray Photoelectron Spectroscopy

A spider was euthanized and hemolymph samples were collected from the ventral side of the spider between the sternum and petiole following a procedure established in prior work.^[^ ^83^ ^]^ The hemolymph was carefully spread on pristine silicon wafer sections to avoid any unknown sources of contamination that could skew the X‐ray photoelectron spectroscopy (XPS) survey spectra. Tweezers were used to remove the legs of the spider and the samples were allowed to dry for an hour. The XPS was carried out using a PHI Quantera SXM scanning X‐ray microprobe with a conventional Al K*α* (1486.6 eV) X‐ray source. Survey scans were recorded at 140 eV pass energy, and high‐resolution scans were obtained at 26 eV pass energy. The atomic elemental composition analyses and curve fitting were performed using Multipak XPS software.

### Force versus Displacement Characterization

A universal testing machine (Instron, 86SC‐2) was used to precisely control the displacement and the rate at which the necrobotic gripper was raised. An analytical balance (Mettler Toledo, ME204E) was used to determine the gripping force. Because the gripping forces were on the order of 100 µN and the gripper shape was irregular, we used a customized “weight” made of a cellulose acetate bead, a dispensing needle, and a heavy acrylic base. The bead was chosen as the point of gripping to accommodate the radial inward flexion of the gripper, the needle was used to suspend the bead to avoid any obstruction, and the acrylic base was used to provide stability. The setup of the experiment is illustrated in Figure S2 (Supporting Information). This custom weight was placed on the analytical balance and the balance was zeroed before the experiment began. A camera was used to record the screens of both the universal testing machine and the analytical balance, which display the displacement and the mass lifted by the gripper, respectively. The analytical balance has a resolution of 0.1 mg (≈0.001 mN), which is suitable to characterize the gripping force (≈0.01 mN or greater). A digital pressure gauge was used to monitor the pressure used to actuate the necrobotic gripper. Because the necrobotic gripper is exerting a lifting force on the weight, the magnitude of the resulting negative value shown on the analytical balance corresponds to the gripping force exerted by the necrobotic gripper. The resulting data for the force versus displacement curves shown in Figure 2b of the main text were extracted manually from the video recordings of the experiment, and mass was converted to force.

## Conflict of Interest

The authors declare no conflict of interest.

## Author Contributions

T.F.Y. and D.J.P. conceived the research. T.F.Y., Z.L., A.R., and D.J.P. designed the experimental setups and collected data. T.F.Y. and D.J.P. analyzed the data. Z.L. and T.J.S. conducted SEM imaging to visualize the spider joints and seal at the insertion point. T.F.Y., T.J.S., and D.J.P. contributed to writing the original draft. All authors contributed to writing and reviewing the final draft, and have given their consent to the final version of the manuscript. D.J.P. supervised the research.

## Supporting information

Supporting Information

Supplemental Movie 1

Supplemental Movie 2

Supplemental Movie 3

Supplemental Movie 4

## Data Availability

The data that support the findings of this study are available in the supplementary material of this article.
