# Supplementary material for: Necrobotics: Biotic Materials as Ready‐to‐Use Actuators
Source: Adv Sci (Weinh). 2022 Jul 25;9(29):2201174. doi: 10.1002/advs.202201174 (PMC9561765; doi:10.1002/advs.202201174)
Supplement: Supplementary file 1 — Supporting Information [file ADVS-9-2201174-s002.pdf]

ADVANCED SCIENCE
Open Access

Supporting Information

for *Adv. Sci.*, DOI 10.1002/advs.202201174

Necrobotics: Biotic Materials as Ready-to-Use Actuators

*Te Faye Yap, Zhen Liu, Anoop Rajappan, Trevor J. Shimokusu and Daniel J. Preston**

Supporting Information

Necrobotics: Biotic Materials as Ready-to-Use Actuators

*Te Faye Yap, Zhen Liu, Anoop Rajappan, Trevor J. Shimokusu, Daniel J. Preston**

T. F. Yap, Z. Liu, Dr. A. Rajappan, T. J. Shimokusu, Prof. D. J. Preston

Department of Mechanical Engineering, Rice University, 77005, USA

E-mail: djp@rice.edu

1. Fabrication of Necrobotic Grippers

The necrobotic gripper was fabricated by inserting a hypodermic needle into a deceased spider (Figure S1). We purchased a wolf spider (of the family *Lycosidae*) from a supplier of biological specimens (Carolina Biological Supply), and euthanized it via freezing at a temperature of approximately –4 °C for 5–7 days. After the spider died, we stabilized the deceased spider with tweezers and inserted a 25-gauge Luer lock hypodermic needle into the prosoma of the spider, ensuring that the tip of the needle was fully inserted into the body. We used all-purpose cyanoacrylate glue (KG585, "Krazy Glue") to secure and seal the needle at the insertion point. We applied a drop of glue onto the needle and allowed it to flow toward the needle-spider interface, where the glue formed a meniscus surrounding the needle to create a hermetic seal; we describe this "self-sealing" mechanism in detail in the main text. We allowed the glue to dry for 10 minutes, after which the necrobotic gripper fabrication process was completed.

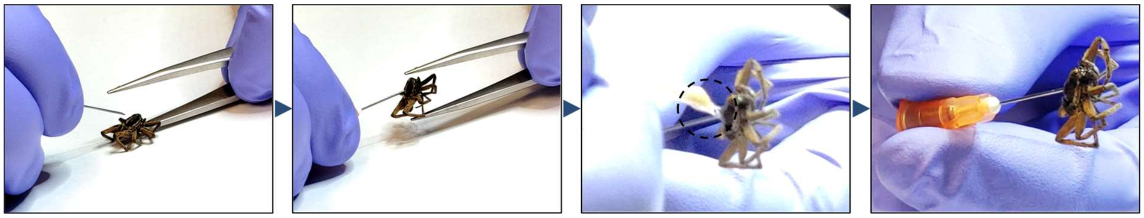

Figure S1. The necrobotic gripper fabrication process is demonstrated with a sequence of images captured during fabrication. From left to right: stabilization of the deceased spider, insertion of the needle, application of glue, and curing of glue.

2. Experimental Setup to Determine Force versus Displacement

A universal testing machine (Instron, 86SC-2) and an analytical balance (Mettler Toledo, ME204E) were used to precisely control the displacement and quantify the gripping force as detailed in the *Experimental* section of the main text and shown in Figure S2.

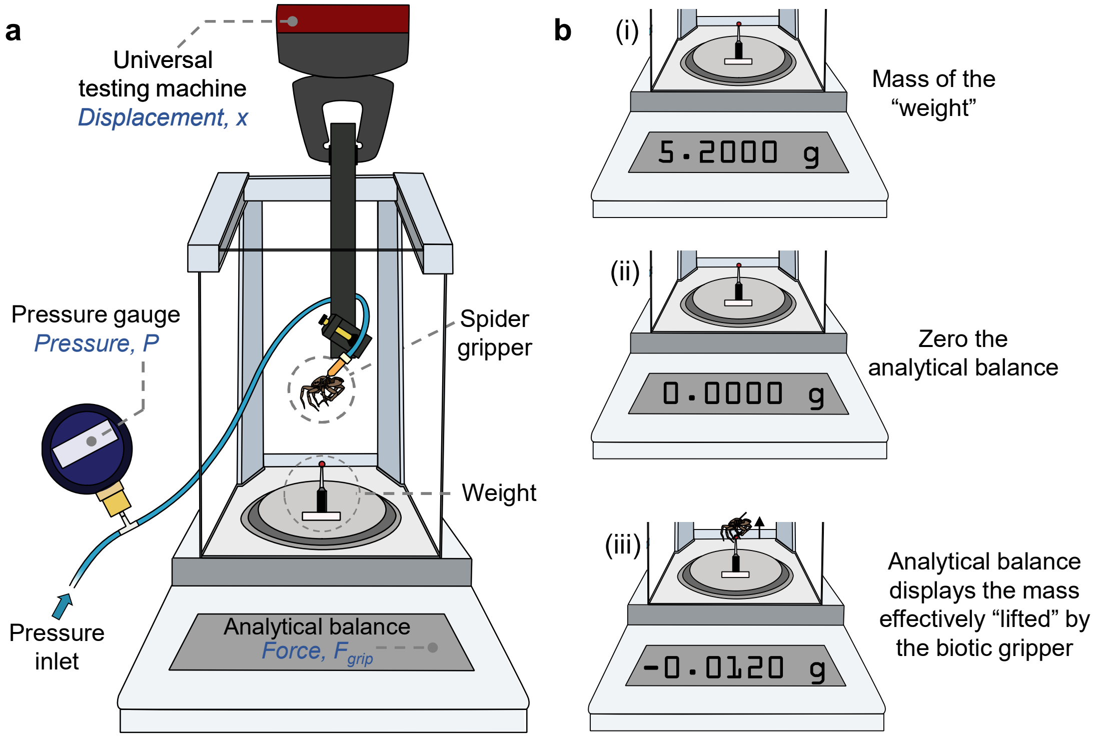

Figure S2. (a) Illustration of the experimental setup for force versus displacement characterization shown in Figure 2 of the main text. (b) The process in which we conduct the experiment to determine the gripping force of the necrobotic gripper. (b-i) We place the custom weight on the analytical balance. (b-ii) We tare the analytical balance to negate the mass of the weight. (b-iii) When the necrobotic gripper grips onto the weight and is raised, the magnitude of the analytical balance reading is the mass effectively lifted by the necrobotic gripper, which is converted to the gripping force.

3. SEM Images of Spider Setae

Motivated by our discussion in the main text of setae (small hair-like structures on the spider) potentially providing additional adhesion force, we visualized the geometry of setae present on this spider using scanning electron microscopy (Figure S3). The spider leg was first sputtered with a 10-nm-thick titanium coating that acts as an adhesion layer and subsequently sputtered with a 20-nm-thick coating of gold to improve the conductivity of the spider surface. The sputtering processes was conducted with an AJA ATC Orion Sputtering System (load-locked, 8-gun magneton sputtering system) using direct current (DC) plasma at the maximum power of 50W. A field emission scanning electron microscope (FEI Quanta 400 ESEM FEG) was then used to take images with the secondary electron detector at an accelerating voltage of 10 kV. We were also able to capture the morphology of the nanostructures present at the joint membrane, which has been observed in prior work.[1]

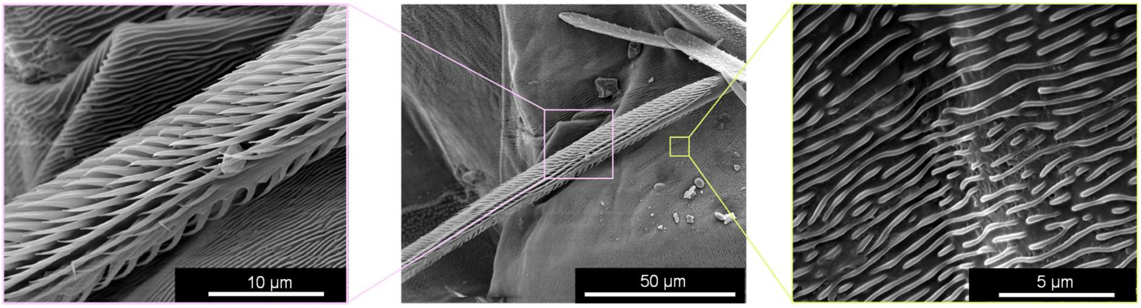

Figure S3. Scanning electron microscope images of the setae (spider hair) structure at left and center, and nanostructures present on the spider cuticle surface at right.

4. Ability to Camouflage

Because spiders have physical appearances that allow them to blend in with their natural environment, the necrobotic gripper is able to camouflage and will potentially be able to perform tasks unnoticed. The pressure source could be modified to involve a device that can be controlled from a remote location. For example, using a transparent length of tubing that is connected to the necrobotic gripper could enable long range applications. We demonstrate the necrobotic gripper's camouflaging capabilities by using a handheld actuation mechanism in an outdoor environment, showing the necrobotic gripper grasping a leaf while appearing almost unnoticeable to the human eye. This characteristic of the necrobotic gripper is useful for applications that require a discreet way of collecting delicate samples in nature.

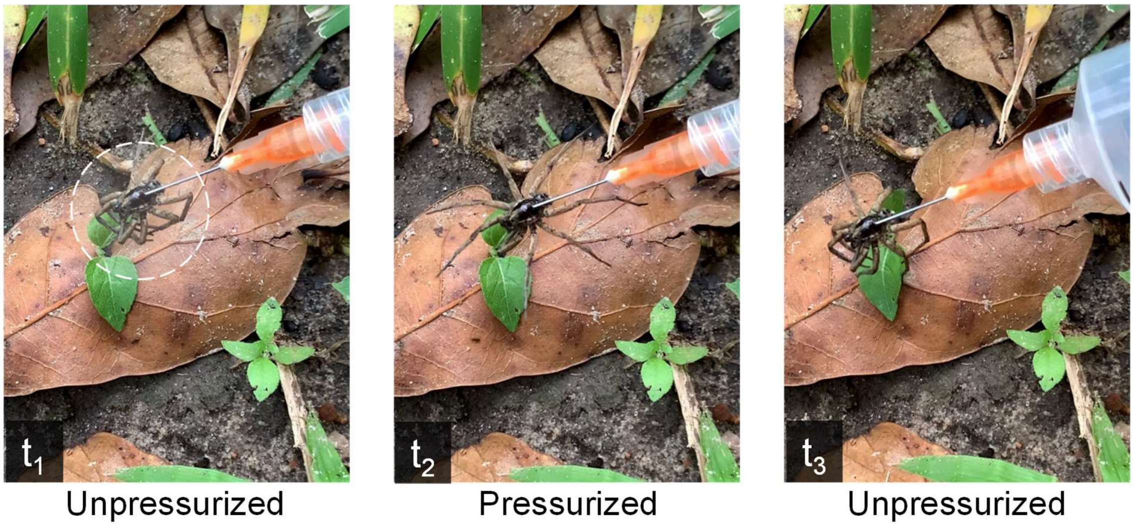

Figure S4. A time-lapse sequence of photos showing the untethered actuation mechanism of the necrobotic gripper in an outdoor environment. The spider is able to blend in with the background, a potentially useful characteristic for field research which requires a non-disruptive manner of collecting samples in nature. The necrobotic gripper is highlighted in the first image.

5. Degradation of Spider Joints After Cyclic Testing

To illustrate the mode of degradation of the patellofemoral joint after 1000 cycles of actuation, we compared the physical morphology of the joint prior to cyclic testing (Figure S5a) and after cyclic testing (Figure S5b-c). We were able to compare the legs from a similar spider by carefully removing one of the legs prior to cyclic testing, and using superglue to seal the cavity to prevent leaks and continue to be able to operate the gripper. After performing the cyclic testing, we observed micron-sized cracks on the surface of the articular membrane, which could explain the permanent deformation of the articular membrane at the patellofemoral joint angle observed in Figure 3c. The formation of these cracks could be due to dehydration of the thin articular membrane which resulted in the membrane becoming more brittle and susceptible to mechanical fracture. Application of thin, flexible coatings could circumvent this issue by delaying or preventing dehydration through the exoskeleton (see Section 7 in Supporting Information).

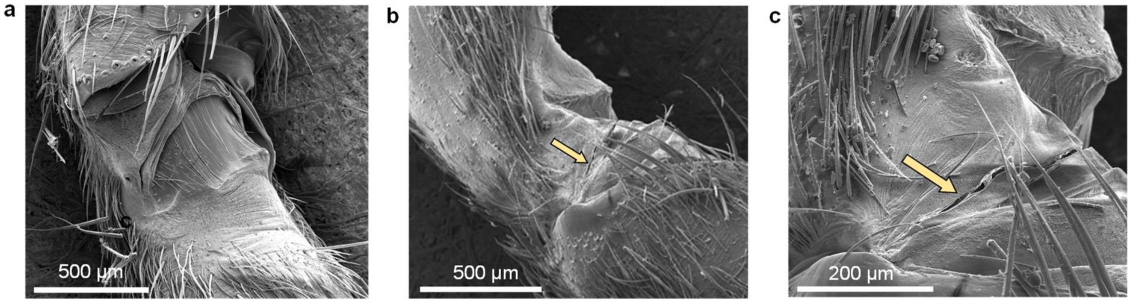

Figure S5. (a) Scanning electron microscopy (SEM) image of patellofemoral joint from the necrobotic gripper prior to cyclic testing. (b) SEM image of patellofemoral joint after cyclic testing. (c) Magnified SEM image of a crack on the order of 100 µm (indicated by the arrows) observed on the articular membrane.

6. Chemical Composition of Spider Leg and Hemolymph

We characterized the chemical composition of the spider's leg and hemolymph for two cases, (i) one hour after euthanasia and (ii) seven days after euthanasia, using X-ray photoelectron spectroscopy (XPS). We euthanized a spider and collected hemolymph samples from the ventral side of the spider between the sternum and petiole following a procedure established in prior work.[2] We prepared two samples with each of the legs and hemolymph for comparison of cases (i) and (ii) using XPS. We prepared the hemolymph sample by spreading the hemolymph on two pristine silicon wafers to eliminate any unknown sources of contamination that could skew the XPS survey spectra. We carefully removed the legs of the spider to characterize the change in chemical composition of the legs before and after exposure to the ambient environment.

The XPS characterization was performed using a PHI Quantera SXM scanning X-ray microprobe with a conventional Al Kα (1486.6 eV) X-ray source. Survey scans were recorded at 140 eV pass energy, and high-resolution scans were obtained at 26 eV pass energy. We performed the atomic elemental composition analyses and curve fitting using Multipak XPS software. Figure S6 shows the elemental composition of the leg and the hemolymph one hour after the spider was euthanized and seven days after its death. Figures S7 and S8 show the high-resolution XPS spectra and curve fitting results for the spider's legs and hemolymph, respectively.

Carbon and oxygen were detected on the leg exoskeleton in both cases, with an atomic carbon percentage of 94.6% and an atomic oxygen percentage of 5.4% for case (i) and an atomic carbon percentage of 92.9% and an atomic oxygen percentage of 7.1% for case (ii). The elemental percentage difference between the two cases is less than 2%, indicating a negligible change in chemical composition of the spider's leg seven days after euthanasia. The curve fitting results from the high-resolution spectra of the spider's legs (Figure S7) indicate that

carbon sp^2, C-O (from both C-OH and C-O-C), and N-C=O chemical bonds are present on the leg. This chemical bonding composition agrees well with the chemical composition of chitin, which is a primary component of the exoskeleton of arthropods.[3] The percentage difference of each chemical bond observed here for the two cases is less than 6%, indicating no major changes in chemical bonds for the spider's leg seven days after death. The slight increase in oxygen composition might be due to mild oxidation of the exoskeleton exposed to the ambient.

As for the hemolymph, carbon, oxygen, nitrogen, sodium, and chlorine were detected from the survey spectra (Figure S6b), all of which are expected to be present due to the inorganic salts and organic compounds (e.g., carbohydrates, proteins, and lipids) found in hemolymph.[4] Similar to the results obtained for the leg, the difference in the percentage of the elemental composition and the chemical bonds (Figure S8) for the two cases are within 6%. This difference could be caused by variations in the chemical composition which depend on the specific location of XPS scanning or other measurement uncertainties. In summary, from both the elemental and chemical bonding analyses, no major changes in chemical composition were found for the spider's leg or hemolymph seven days after euthanasia as compared to the as-euthanized spider.

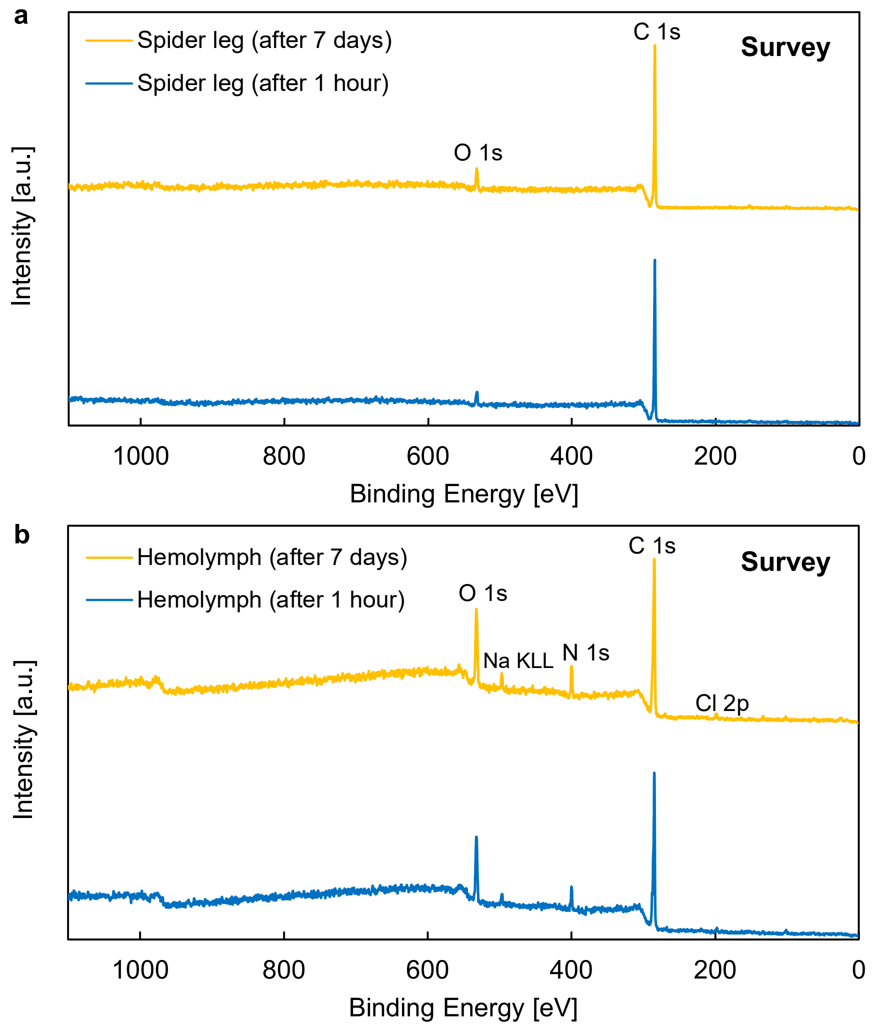

Figure S6. Survey XPS spectra of spider's (a) legs and (b) hemolymph one hour after being euthanized and seven days after death.

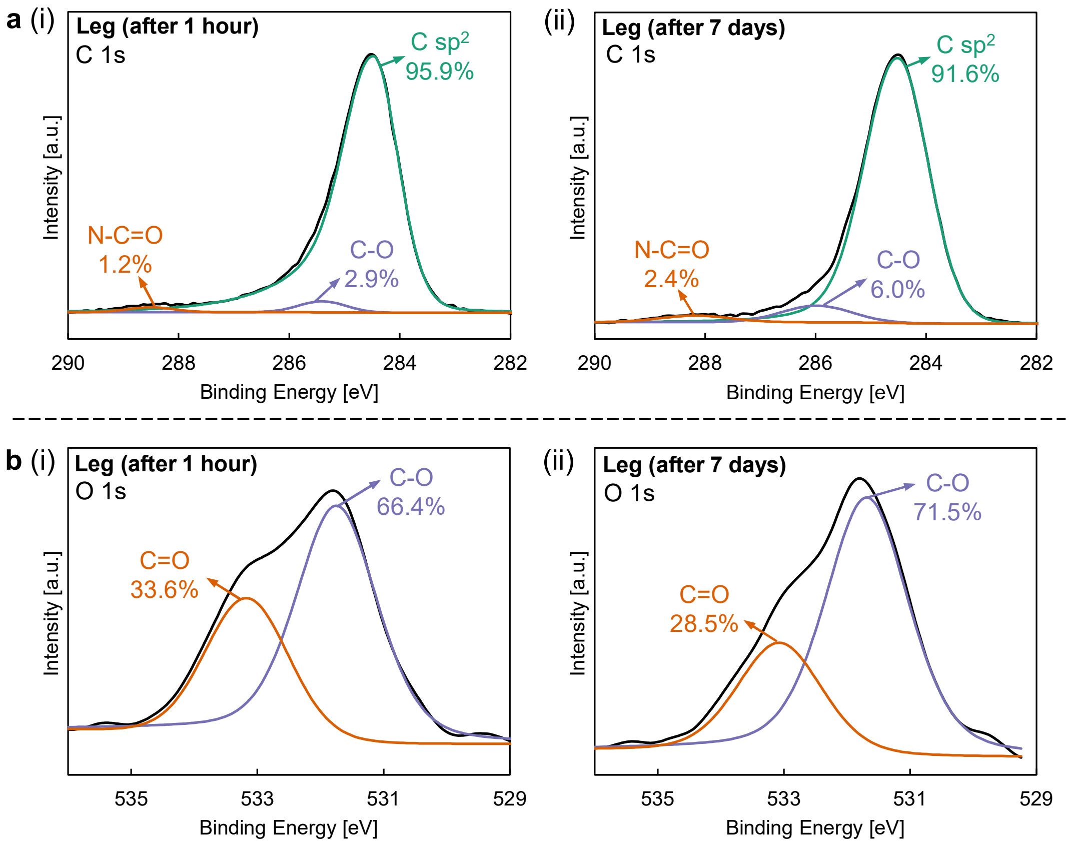

Figure S7. High-resolution XPS spectra of the spider's legs. (a-i) C 1s spectra one hour after euthanasia; (a-ii) C 1s spectra seven days after death; (b-i) O 1s spectra one hour after euthanasia; (b-ii) O 1s spectra seven days after death

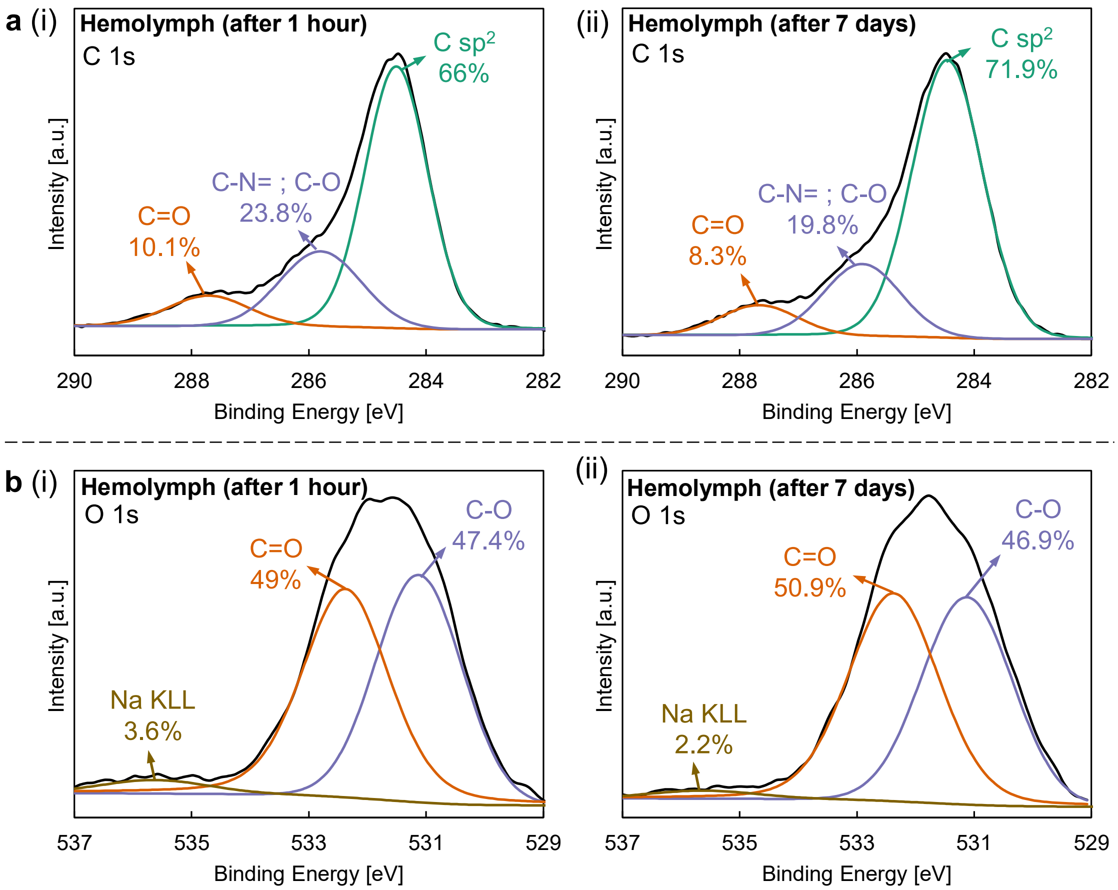

Figure S8. High-resolution XPS spectra of a spider's hemolymph. (a-i) C 1s spectra one hour after euthanasia; (a-ii) C 1s spectra seven days after death; (b-i) O 1s spectra one hour after euthanasia; (b-ii) O 1s spectra seven days after death.

7. Application of Coatings

To prevent dehydration, and ultimately brittle fracture, of the articular membrane, a layer of vapor-impermeable coating could be applied to prolong the lifetime of the necrobotic gripper. We performed preliminary experiments by coating an inanimate spider with a layer of beeswax and measured the change in mass over 10 days. We compared the change in mass with an uncoated inanimate spider as the control. From our results, we show that by coating the spider, we are able to circumvent significant loss in mass, Δm (which we attribute to the loss in water mass), as compared to the uncoated spider (Figure S9). We measured the mass of the spider using an analytical balance (Mettler Toledo, ME204E) with a resolution of 0.1 mg and showed that the uncoated spider experienced a 72.03% decrease in mass while the coated spider had only a 0.69% decrease in mass after exposure to the lab environment (average temperature and relative humidity of 23 °C and 47%) for 10 days. This initial result warrants future investigation of different conformable and flexible vapor-impermeable coating materials to mitigate water loss via evaporation from the exoskeleton while maintaining the operability of the necrobotic gripper.

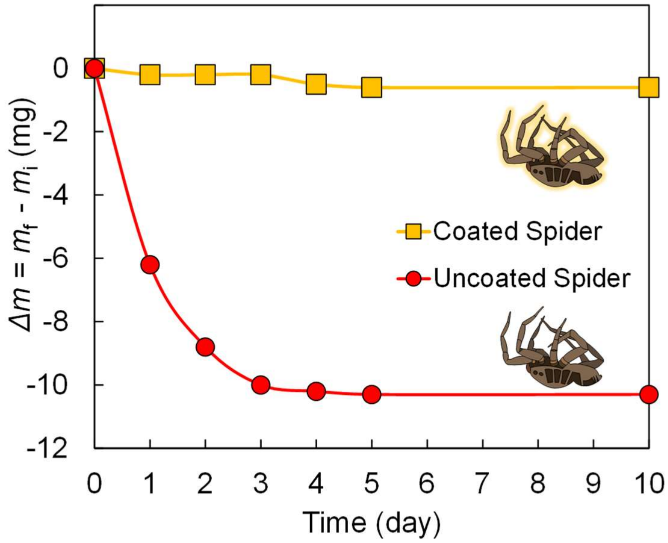

Figure S9. Plot showing the change in mass, Δm, for a spider coated with beeswax versus an uncoated spider over a period of 10 days.

8. Effects of Object Size on Gripping Force

To illustrate the effects of varying geometric parameters on the maximum gripping force, we repeated the experiment shown in Figure 2a and Figure S2 with three acetate beads of different diameters: (i) 3.03mm (used in Figure 2a), (ii) 4.76 mm, and (iii) 6.20 mm. The results show that the maximum gripping force increases with the diameter of the bead. We observed that the variation in maximum gripping force remains within an order of magnitude (~ 1 mN), and consequently this variation in maximum gripping force does not significantly impact the scaling analysis.

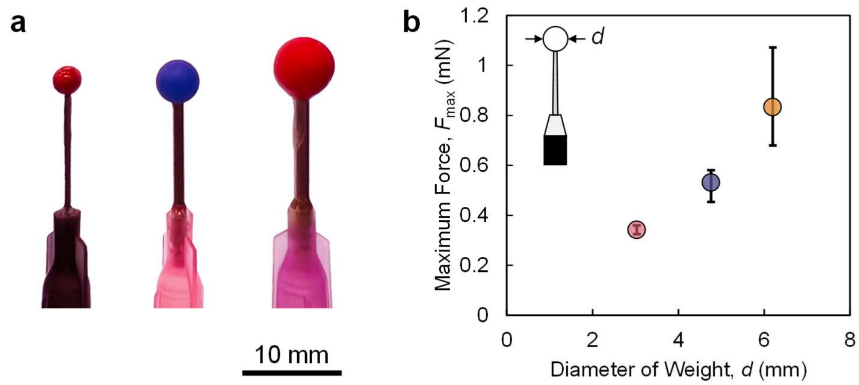

Figure S10. (a) Photograph of the "weights" with different diameters used for the experiment. (b) Data for the maximum gripping force (average of three trials) of the necrobotic gripper for different diameters. The error bars show the range (maximum and minimum) of gripping forces attained by the necrobotic gripper.

Supporting Information References

[1] C. Göttler, K. Elflein, R. Siegwart, M. Sitti, *Adv. Sci.* **2021**, *8*, 2003890.
[2] C. Göttler, G. Amador, T. van de Kamp, M. Zuber, L. Böhler, R. Siegwart, M. Sitti, *Soft Matter* **2021**, *17*, 5532.
[3] D. Elieh-Ali-Komi, M. R. Hamblin, **2017**, 24.
[4] A. D. Sowers, S. P. Young, M. Grosell, C. L. Browdy, J. R. Tomasso, *Comparative Biochemistry and Physiology Part A: Molecular & Integrative Physiology* **2006**, *145*, 176.

Other supporting information for this manuscript includes the following:

Movie S1. Breaking a circuit. Demonstration showing the necrobotic gripper breaking a closed circuit by removing a jumper wire.

Movie S2. Gripping an irregular object of larger mass. Demonstration of the ability of the necrobotic gripper to grasp a spider with an irregular geometry and a larger mass.

Movie S3. Picking up an object of larger volume Demonstration of the ability of the necrobotic gripper to pick up a colored foam block of larger volume.

Movie S4. Untethered operation of the necrobotic gripper. Demonstration of an untethered actuation scheme for the necrobotic gripper achieved by attaching a handheld syringe.
